# Research on the Stability of a Rabbit Dry Eye Model Induced by Topical Application of the Preservative Benzalkonium Chloride

**DOI:** 10.1371/journal.pone.0033688

**Published:** 2012-03-16

**Authors:** Chaoyang Li, Yiyue Song, Shaohong Luan, Pengxia Wan, Naiyang Li, Jing Tang, Yu Han, Cuiju Xiong, Zhichong Wang

**Affiliations:** 1 State Key Laboratory of Ophthalmology, Zhongshan Ophthalmic Center, Sun Yat-sen University, Guangzhou, China; 2 Zhongshan Ophthalmic Center, Sun Yat-sen University, Guangzhou, China; 3 Department of Ophthalmology, People's Hospital of Leshan, Leshan, China; University of Florida, United States of America

## Abstract

**Background:**

Dry eye is a common disease worldwide, and animal models are critical for the study of it. At present, there is no research about the stability of the extant animal models, which may have negative implications for previous dry eye studies. In this study, we observed the stability of a rabbit dry eye model induced by the topical benzalkonium chloride (BAC) and determined the valid time of this model.

**Methods and Findings:**

Eighty white rabbits were randomly divided into four groups. One eye from each rabbit was randomly chosen to receive topical 0.1% BAC twice daily for 2 weeks (Group BAC-W2), 3 weeks (Group BAC-W3), 4 weeks (Group BAC-W4), or 5 weeks (Group BAC-W5). Fluorescein staining, Schirmer's tests, and conjunctival impression cytology were performed before BAC treatment (normal) and on days 0, 7, 14 and 21 after BAC removal. The eyeballs were collected at these time points for immunofluorescence staining, hematoxylin and eosin (HE) staining, and electron microscopy. After removing BAC, the signs of dry eye in Group BAC-W2 lasted one week. Compared with normal, there were still significant differences in the results of Schirmer's tests and fluorescein staining in Groups BAC-W3 and BAC-W4 on day 7 (*P<0.05*) and in Group BAC-W5 on day 14 (*P<0.05*). Decreases in goblet cell density remained stable in the three experimental groups at all time points (*P<0.001*). Decreased levels of mucin-5 subtype AC (MUC5AC), along with histopathological and ultrastructural disorders of the cornea and conjunctiva could be observed in Group BAC-W4 and particularly in Group BAC-W5 until day 21.

**Conclusions:**

A stable rabbit dry eye model was induced by topical 0.1% BAC for 5 weeks, and after BAC removal, the signs of dry eye were sustained for 2 weeks (for the mixed type of dry eye) or for at least 3 weeks (for mucin-deficient dry eye).

## Introduction

Dry eye is a common disease worldwide [1]. Clinical epidemiological surveys have demonstrated that in 1995, 17% of the outpatients in Japan reported symptoms of dry eye [2], and in 1997, 14.6% of the U.S. population who ranged in age from 65 to 84 years showed symptoms of dry eye [3]. In 2003, approximately 33.7% of the elderly population of Taiwan (those over the age of 65 years) also reported symptoms of dry eye [4]. The high prevalence of dry eye has already evoked the close attention of scholars from all over the world, so there has been a great advancement in dry eye research over the last decade. Dry eye has been redefined in a report from the Dry Eye WorkShop (DEWS), which summarized the results of these studies. Increased osmolarity of the patient's tears and inflammation of the ocular surface were added to the new definition, and the potential damage to the ocular surface was clearly indicated [5]. However, there is still much to be learned from the study of the dry eye disease because dry eye is a multifactorial and complex disease.

Standard experimental animals are primary agents that directly impact the accuracy of experimental results. The investigation of dry eye is imperative for determining both its causes and appropriate treatments for it, and several animal models have been created. For example, Susi built a rabbit model of dry eye using a topical administration of 1% atropine and observed the model for 6 days [6]. Barabino constructed a murine dry eye model that used a controlled environment of temperature, humidity, and wind velocity to induce the condition; this model was observed for 28 days [7]. In his subsequent experiments, however, he only placed the mice in this environment for 3 or 7 days [8], [9]. Dursun built a dry eye model in Sprague-Dawley (SD) rats by applying transdermal scopolamine and placing the model animals in a desiccating environment for 4 days [10]; in other experiments, these authors used a number of different treatment durations (4 hours, 1 day, 2 days, 3 days, 5 days, 10 days, 12 days and so on) to validate their model according to various detection indices of dry eye [11]–[14]. Wei Chen et al. established a murine dry eye model that used an intelligently controlled environmental system for 42 days to establish the condition [15]. In subsequent experiments, they chose to treat the mice for 21 days [16]. The current authors have previously built a rabbit model of dry eye using a topical application of 0.1% benzalkonium chloride (BAC) for 2 weeks [17]. In addition to the aforementioned studies, many other methods of building animal models of dry eye have been reported [18]–[23].

When we used our rabbit dry eye model for the experiment that is described in this article, we found that some indices of the rabbit models showed that the animals tend to recover within two weeks of successfully establishing the dry eye model. This phenomenon suggested that this model of dry eye was not stable. After reviewing the literature, we found that all of the standards for evaluating dry eye models have depended solely on signs of dry eye that are detected when the animal models were correctly created, and we discovered that no one had conducted follow-up research on these models [6], [7], [10], [15].

As a result of this discovery, we designed this experiment to observe the stability of a rabbit model of dry eye following the cessation of regular BAC administration; to be more specific, we decided to observe the duration over which dry eye signs were maintained in rabbits after successfully building the model, and in doing so, we determined the temporal validity of this model. The results of this experiment would both validate this model of dry eye and determine whether it is a reliable tool for dry eye research.

## Materials and Methods

### Animals and Experimental Procedure

All of the procedures in this experiment were performed in accordance with the ARVO Statement for the Use of Animals in Ophthalmic and Vision Research and were approved by the animal ethics committee of Zhongshan Ophthalmic Center, Sun Yat-sen University (approval ID: 2008-027). Eighty female New Zealand white rabbits (2–2.5 kg, purchased from the Guangdong Medical Laboratory Animal Center, Guangdong, China) were used for this study. The environmental standards throughout this study were the same as in our previous study [17]; rabbits were housed at a room temperature of 23°C±2°C with a relative humidity 60%±10%, and alternating 12-h light-dark cycles (8 A.M. to 8 P.M.). The eighty rabbits were randomly divided into four groups (BAC-W2, BAC-W3, BAC-W4 and BAC-W5), and one eye from each rabbit was randomly chosen as the experimental eye. This eye was treated with a twice-daily (8 A.M. and 4 P.M.) topical administration of 0.1% BAC (Sigma, St. Louis, MO, USA). Group BAC-W2 was medicated for 2 weeks, Group BAC-W3 was medicated for 3 weeks, Group BAC-W4 was medicated for 4 weeks, and Group BAC-W5 was medicated for 5 weeks. Fluorescein staining, Schirmer's tests, and the conjunctival impression cytology were performed sequentially following the methods described below. These tests were performed prior to treatment (normal), immediately after the cessation of the BAC administrations (day 0) and 1 week (day 7), 2 weeks (day 14), and 3 weeks (day 21) after stopping the BAC treatment. Before beginning the BAC treatment, one rabbit from each group was randomly chosen to be sacrificed, and the ocular global tissues from each of these animals were carefully dissected and prepared for immunofluorescence staining, hematoxylin and eosin (HE) staining, and scanning and transmission electron microscopy according to the methods that are described below. In addition, two rabbits from each group were randomly chosen on days 0, 7, 14 and 21 after the cessation of BAC treatment and were sacrificed, after which the ocular global tissues from these animals were dissected and prepared for staining and microscopy.

### Measurement of Aqueous Tear Secretion

Aqueous tear secretion was measured with a modified Schirmer I test using strips of Whatman 41 filter paper (Tianjin Jingming New Technology Development Co. Ltd., Tianjin, China). Tests were conducted at the same time points on prearranged days in the standard environment by the same person. Intramuscular injections of a mixture of 25 mg ketamine and 25 mg chlorpromazine were administered to keep the rabbits immobile. After a topical administration of Alcaine (Alcon, Fort Worth, TX, USA), we pulled the lower eyelid down slightly and placed the paper strip for the Schirmer's test on the palpebral conjunctival vesica, which is near the junction of the middle and outer third of the lower lid. After 5 min, the wetted length (in millimeters) of the paper strip was read. Each eye was tested 3 times, and the average length of wetted paper was taken as the final length.

### Fluorescein (FL) Staining

Two microliters of a 1% sodium fluorescein solution (Zhongshan Ophthalmic Center, Guangzhou, China) were introduced into the conjunctival sac; 2 minutes after instillation, any superfluous sodium fluorescein sodium was washed out with a normal saline solution. The staining was then examined under a slit lamp microscope with a cobalt blue filter (Topcon, Tokyo, Japan) and recorded. The cornea was divided into four quadrants, each of which was scored as follows: no staining was given a score of 0; slight, punctate staining of fewer than 5 spots was given a score of 1; punctate staining of more than 5 spots with no positive plaque was given a score of 2; and the presence of a positive fluorescein plaque was given a score of 3. The four scores were then added to calculate the final grades, which had totals that were between 0 and 12. This procedure was always performed by the same person.

### Conjunctival Impression Cytology (CIC)

Following an intramuscular injection of a mixture of 25 mg ketamine and 25 mg chlorpromazine, 2 3.5 mm×3.5 mm circular disks of nitrocellulose filter paper (Pall, New York, NY, USA) were placed separately on the nasal and temporal bulbar areas of the conjunctiva with the dull sides of the filter paper down and held in place for 10 seconds under constant pressure. The filter paper was then gently lifted and treated with 95% alcohol. Periodic acid-Schiff (PAS) reagents were used to stain the specimens. Once the papers had been stained, the number of goblet cells in each impression was counted under a microscope (Olympus, Tokyo, Japan) using a 40× objective. Three different sections of each specimen were randomly selected for counting, the number of cells in each section was counted, and an average number of cells was calculated (cells/high-power [HP] visual field at 400×).

### Immunodetection of MUC5AC

Immunodetection of MUC5AC was performed using immunofluorescent staining of cryosections of the nasal and temporal bulbar conjunctiva after the animals had been sacrificed. All specimens were fixed in acetone at 4°C for 10 minutes. After the specimens were washed three times in phosphate buffered saline (PBS), they were blocked with 10% goat serum for 30 minutes at room temperature, then incubated for 12 hours at 4°C with a 1∶100 dilution of mouse anti-human MUC5AC antibody (Abcam, 355660, UK). After 3 more washes in PBS, the specimens were incubated with ALEXA FLUOR secondary goat anti-mouse IgG (Invitrogen, USA) for 30 minutes at room temperature, followed by an additional 3 washes in PBS. Nuclei were counterstained with 0.5 g/mL Hoechst 33342 dye (Invitrogen, USA). Finally, the specimens were observed under a fluorescent microscope (Zeiss, Germany).

### HE Staining of the Cornea

The corneas from sacrificed animals were fixed in 4% formalin for 24 hours after the animals were killed. Briefly, after dehydration, the specimens were embedded in paraffin, cross-sectioned, and stained with hematoxylin and eosin (H&E). The histologic morphology of each corneal specimen was examined using light microscopy.

### Scanning Electron Microscopy (SEM)

Prior to the onset of BAC treatment and on days 0, 7 and 21 after the cessation of BAC treatment, the corneas of animals that had been sacrificed were fixed in a 2.5% glutaraldehyde in 0.1 M phosphate buffer (pH 7.4) for 24 h at 4°C. Specimens were then postfixed with osmium tetroxide in 0.1 M phosphate buffer and dehydrated in a graded series of ethanol solutions. Following dehydration, the fixed specimens were critical-point dried, gold-coated with platinum, and examined with a scanning electronic microscope (JSM-6330F, JEOL, Japan).

### Transmission Electron Microscopy (TEM)

Prior to the start of BAC treatment and on days 0, 7 and 21 following the cessation of BAC treatment, the corneas and conjunctivas of animals that had been sacrificed were fixed via incubation with a 2.5% glutaraldehyde solution in 0.1 M phosphate buffer (pH 7.4) at 4°C for 24 h. They were then postfixed with osmium tetroxide in 0.1 M phosphate buffer, embedded in resin, and cut into 60-nm sections without disturbing the epithelium. The specimens were examined and photographed with a transmission electron microscope (H600, HITACHI, Japan)

### Statistical Analysis

Statistical analyses were performed using the SPSS software package, version 13.0. One-way ANOVAs (Bonferroni) were applied to all of the comparisons of aqueous tear secretion and conjunctival goblet cell density that were made among the groups. A Wilcoxon signed-rank test (Bonferroni) was used for all of the comparisons of FL scores among the groups that were studied. All tests were two-tailed, and P-values that were less than 0.05 were considered to be statistically significant.

## Results

No baseline (normal) significant differences were found among the four experimental groups.

### Aqueous Tear Secretion Experiment

**Figure 1:**
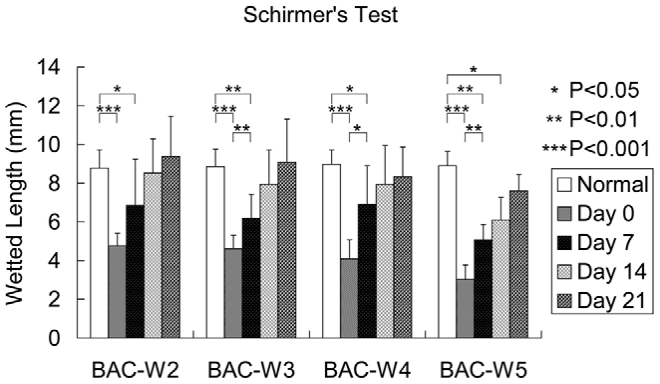
The results of Schirmer's tests in all groups. Schirmer's test results show decreased aqueous tear secretion compared to pre-treatment secretion levels on days 0, 7 and 21 after the cessation of BAC treatment. *P<0.05, **P<0.01, ***P<0.001, respectively. Compared with pre-treatment, the differences were statistically significant on days 0 and 7 in the BAC-W2, BAC-W3, and BAC-W4 groups as well as on days 0, 7 and 14 in Group BAC-W5. The values shown are average wetted lengths ± standard deviations.

Compared with normal eyes, tear secretion declined rapidly in the four groups after BAC treatment. There were statistically significant differences on days 0 and 7 after treatment in Groups BAC-W2, BAC-W3, and BAC-W4, but there were no significant differences on days 14 and 21. However, when comparing tear secretion with the baseline levels in Group BAC-W5, the significant differences lasted until day 14 after the cessation of treatment. No significant differences in the baseline tear secretions of the four groups were recorded prior to the onset of BAC treatment (Fig. 1).

### Corneal Fluorescein Staining

**Figure 2:**
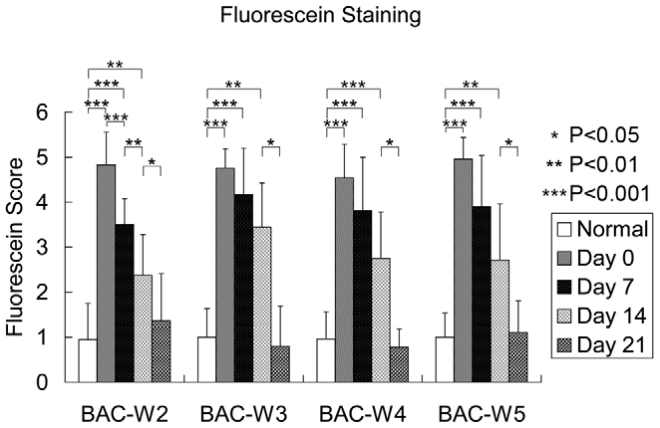
The scores for sodium fluorescein staining in all groups. Compared with the pre-treatment group, there were significant differences on days 0, 7 and 14 after the cessation of BAC treatment in the BAC-W2, BAC-W3, BAC-W4, and BAC-W5 groups.

At baseline, no differences in corneal epithelium fluorescein staining were found among the four groups. Compared with fluorescein staining in normal eyes, the scores for fluorescein staining increased significantly after BAC treatment (*p<0.001*), and these scores for epithelial damage remained significantly higher than normal until day 14 after the cessation of BAC treatment in all of the groups (*p<0.05*) (Fig. 2).

### Conjunctival Impression Cytology and the Density of Goblet Cells

**Figure 3:**
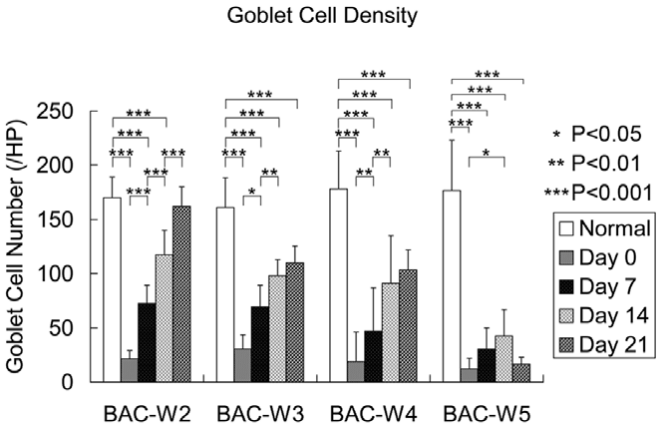
The number of goblet cells in all groups. Compared with the goblet cell densities in normal eyes, there were significant differences on days 0, 7 and 14 in Group BAC-W2, and on days 0, 7, 14 and 21 in Groups BAC-W3, BAC-W4 and BAC-W5. In the last of these (Group BAC-W5), there were no significant differences in goblet cell density between days 0 and 21. The values shown are averages±standard deviations.

**Figure 4:**
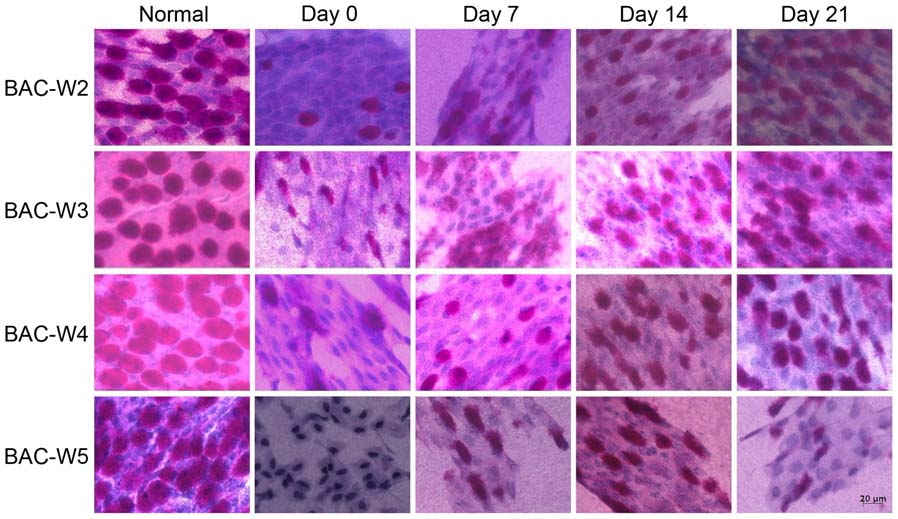
Representative examples of CIC in each group. Each group showed evidence of having an abundance of goblet cells, and the goblet cells were oval in shape and full when normal. On day 0, there were only a few goblet cells in all of the groups, and these goblet cells were irregularly shaped. This difference from baseline could be observed on days 0, 7 and 14 in Group BAC-W2 and on days 0, 7, 14, and 21 in groups BAC-W3, BAC-W4 and BAC-W5.

There were no differences in the goblet cell densities of the four groups at prior to the administration of BAC. The goblet cell densities in all of the BAC-treated groups were significantly reduced on day 0 (*P<0.001*), but the number of goblet cells continued to gradually increase with increases in the amount of time that had elapsed since the cessation of BAC treatment. In Group BAC-W2, this steady increase meant that by day 21, there was no longer a statistically significant difference in the goblet cell density in the eyes of Group BAC-W2 and that of the eyes of the control group. Although the differences in Groups BAC-W3 and BAC-W4 remained significant at all of the time points that were examined, the number of goblet cells in each group tended to recover. However, the trend in Group BAC-W5 was different from that of the first three groups; goblet cell density increased slowly over time until day 14, but on day 21, it decreased to the same level as it had been on day 0 (Fig. 3, Fig. 4).

### Immunofluorescence for MUC5AC

**Figure 5:**
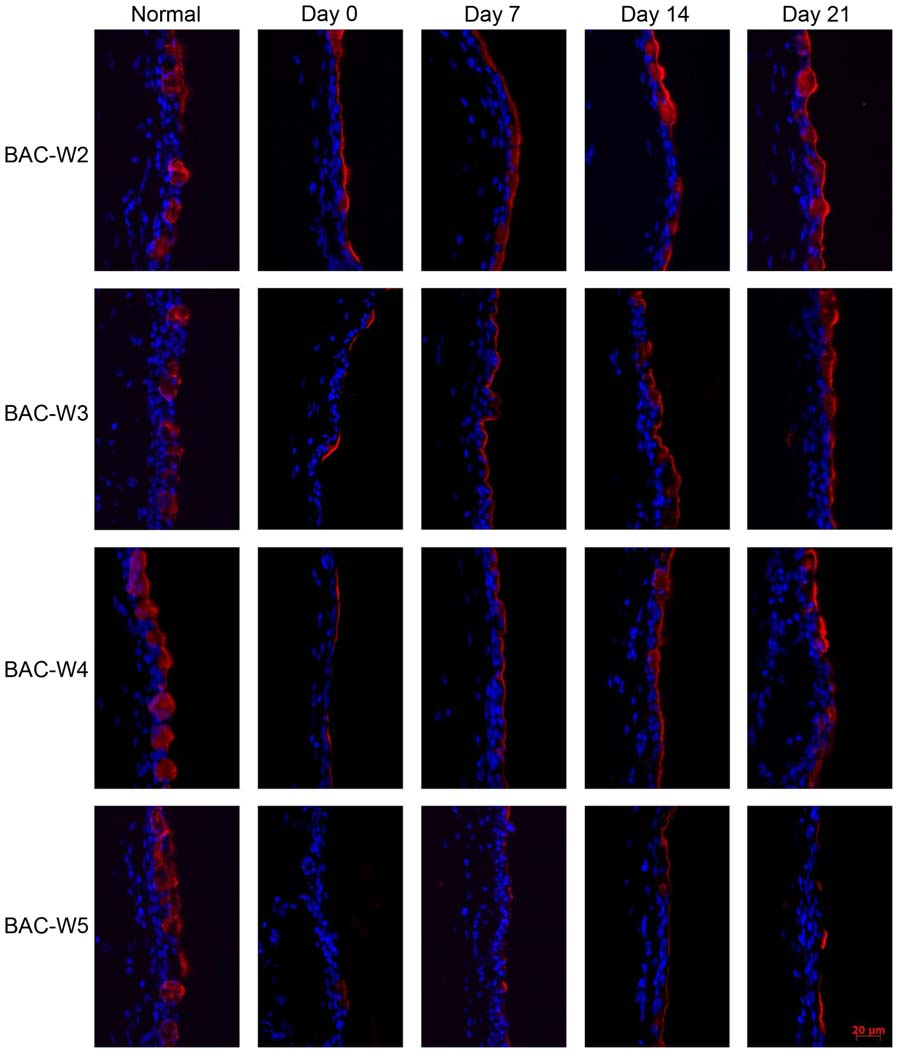
Representative images of immunofluorescence staining in all groups. Prior to treatment, the staining pattern for mucin MUC5AC, which is primarily secreted by the goblet cells, was abundant, and the cells that secrete it were plump and oval in shape. On day 0, the presence of MUC5AC was rare in all of the groups, and the shapes of cells that contained MUC5AC were irregular. This change could also be observed on days 0, 7, 14 in Group BAC-W2; and on days 0, 7, 14 and 21 in Groups BAC-W3, BAC-W4 and BAC-W5.

The changing tendencies of immunofluorescence for MUC5AC in the four groups were consistent with the results of PAS staining (Fig. 5).

### Light Microscopy

**Figure 6:**
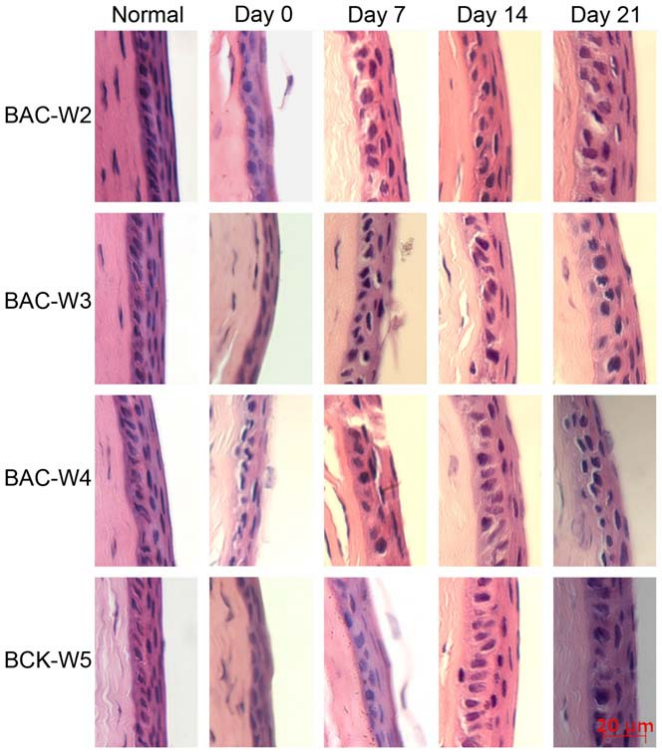
Representative pictures of HE staining of the corneas from each of the four groups. Normal corneas had three to five epithelial layers. Damage to the epithelium could be observed on days 0 and 7 in the four groups.

HE staining of the cornea showed that before BAC treatment, the corneas in all four groups had three to five epithelial layers. In all four groups, the corneal epithelia were thinner on days 0 and 7 than they were prior to the start of treatment, and the cells in them were arranged in a disordered manner. This was especially true for the Group BAC-W5 animals; in these animals, the corneal epithelia became significantly thinner, and in some areas, the epithelial surface cells showed abnormal phenomena (Fig. 6).

### Scanning Electron Microscopy (SEM)

**Figure 7:**
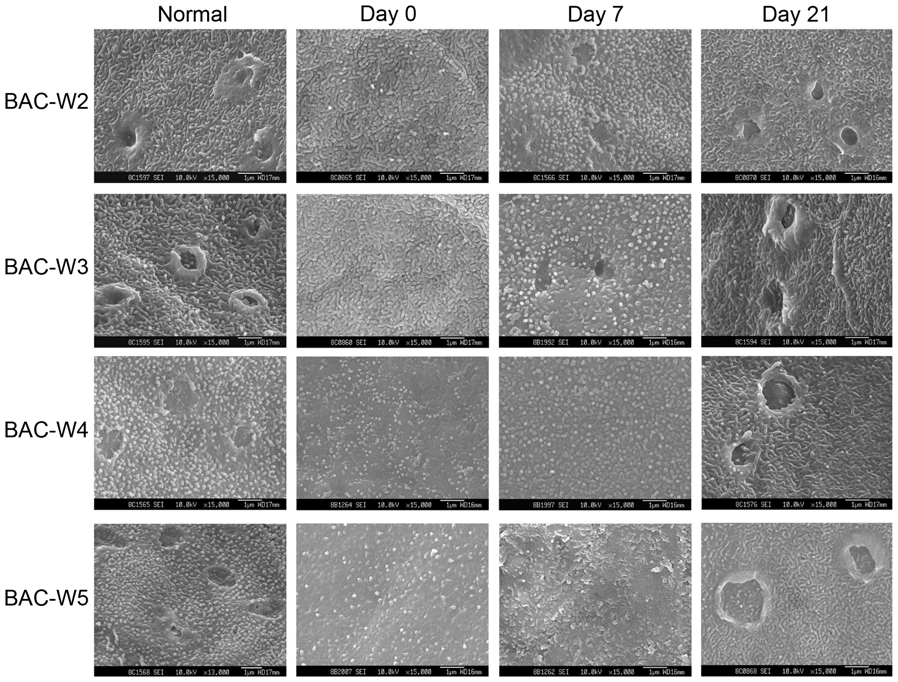
Representative images from scanning electron microscopy. Compared with normal eyes, there were fewer microvilli, and these microvilli were shorter and less regularly arranged on days 0 and 7 in Groups BAC-W2, BAC-W3 and BAC-W4, and on days 0, 7, and 21 in Group BAC-W5.

After the conclusion of BAC treatment, the number of microvilli on the corneal surface decreased significantly, the microvilli became shorter and irregularly arranged, and the micropores on the cell membrane decreased or even completely disappeared. This phenomenon was observable on days 0 and 7 in groups BAC-W2, BAC-W3 and BAC-W4 and on days 0, 7, and 21 in Group BAC-W5. As the amount of time that had elapsed after the cessation of BAC applications increased, the microvilli and micropores of the epithelial cells in Groups BAC-W2 and BAC-W3 gradually returned to normal levels by day 21. The number and morphology of microvilli in Group BAC-W4 recovered to normal on day 21, but the number of micropores was still slightly less than that it was prior to treatment. The numbers of both microvilli and micropores in Group BAC-W5 were still lower on day 21 compared with the numbers of microvilli and micropores in samples from the pre-treatment animals (Fig. 7).

### Transmission Electron Microscopy

#### Corneal Epithelial Ultrastructure

On day 0, necrosis of the corneal epithelium could be observed in each group, especially Group BAC-W5, and the corneal epithelial surface cells were detached in some areas, which resulted in significant levels of intercellular space expansion, many vacuoles between the cells, and organelle damage, such as swelling of the mitochondria, Golgi apparati and the rough endoplasmic reticulum (RER).

**Figure 8:**
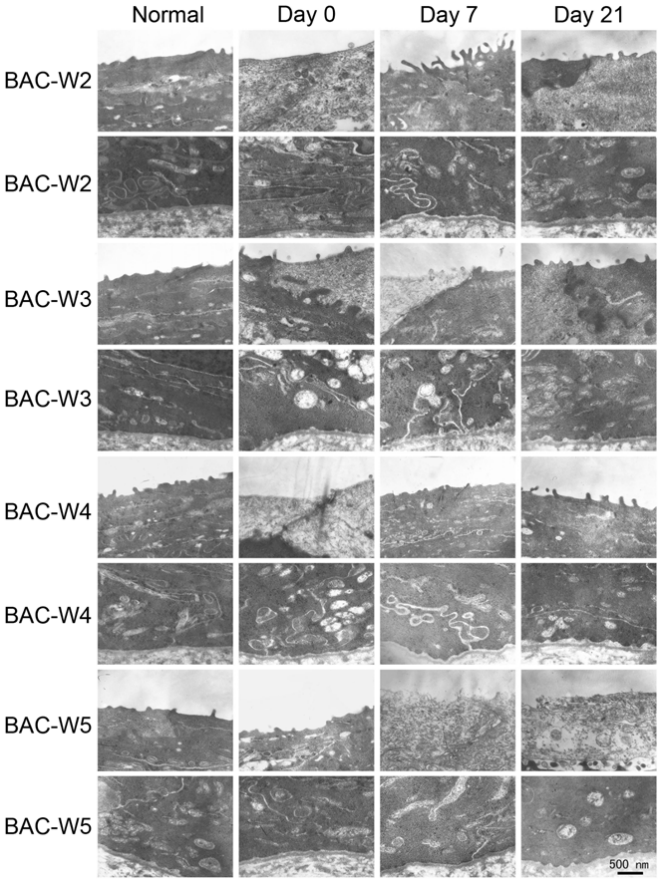
Representative images from transmission electron microscopy. Compared with images from normal eyes, swelling of the mitochondria, Golgi apparati and RER could be observed on day 0 in Group BAC-W2, on days 0 and 7 in Groups BAC-W3 and BAC-W4, and on days 0, 7, and 21 in Group BAC-W5.

**Figure 9:**
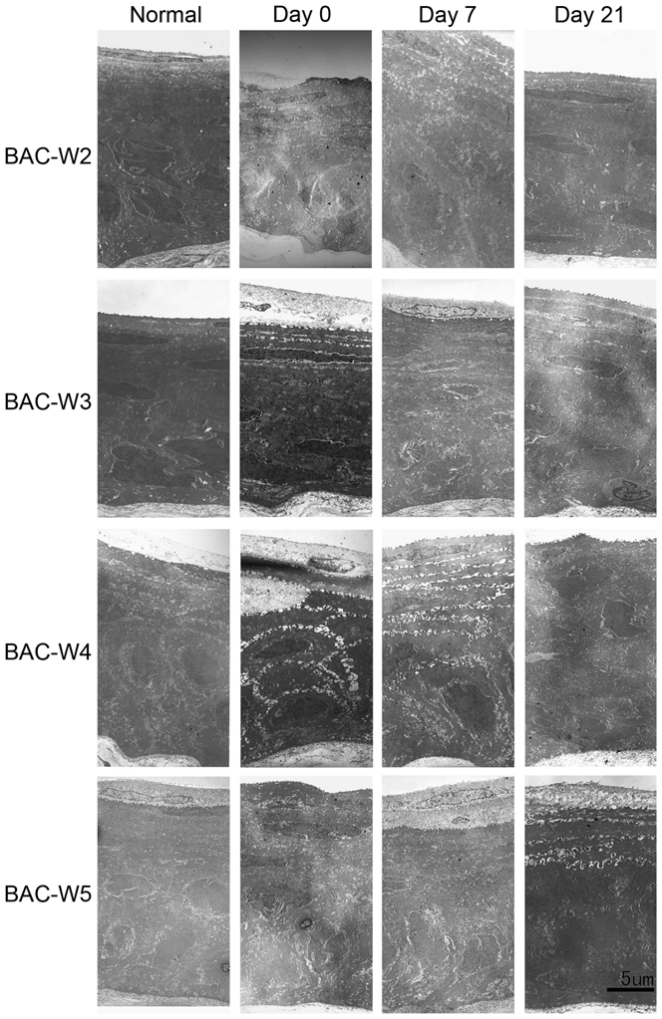
Representative images from transmission electron microscopy that revealed the ultrastructure of the corneal epithelium. Compared with normal eyes, vacuoles between the cells could be observed on day 0 in Group BAC-W2 and on days 0, 7, and 21 in Groups BAC-W3, BAC-W4. These vacuoles were particularly prominent among samples from Group BAC-W5.

The damage that had been caused by treatment with BAC gradually abated in Groups BAC-W2, BAC-W3 and BAC-W4 until day 21; the cellular ultrastructure nearly returned to normal in Groups BAC-W2 and BAC-W3 whereas the situation remained severe in Group BAC-W5 (Fig. 8, Fig. 9).

#### Conjunctival Ultrastructure

By the end of the period in which model animals were treated with BAC, the conjunctival epithelia had become thinner. The goblet cells were in a necrotic state, and we could see that the levels of secretory granules in the goblet cells had either diminished substantially or that the granules had disappeared altogether. Some epithelial cells necrotized, which indicated chromatin margination or cell membrane rupture.

On day 7, the conjunctival epithelia from Group BAC-W2 samples showed evidence of recovery, and on day 21, the conjunctival epithelia were rich in goblet cells and showed the full and normal shapes of secretory granules. Samples from Group BAC-W3 animals exhibited similar trends to those from Group BAC-W2.

In Group BAC-W4, some goblet cells that contained secretory granules could be observed in the conjunctival epithelium on day 7, and on day 21, goblet cells that had abundant numbers of secretory granules in the secretion phase could be found in some areas. However, the goblet cells in other areas remained damaged; in these areas, fewer secretory granules were present, and the areas were not stained as darkly as comparable areas were prior to treatment (presumably because the quality of mucin that was secreted by the goblet cells had degraded).

**Figure 10:**
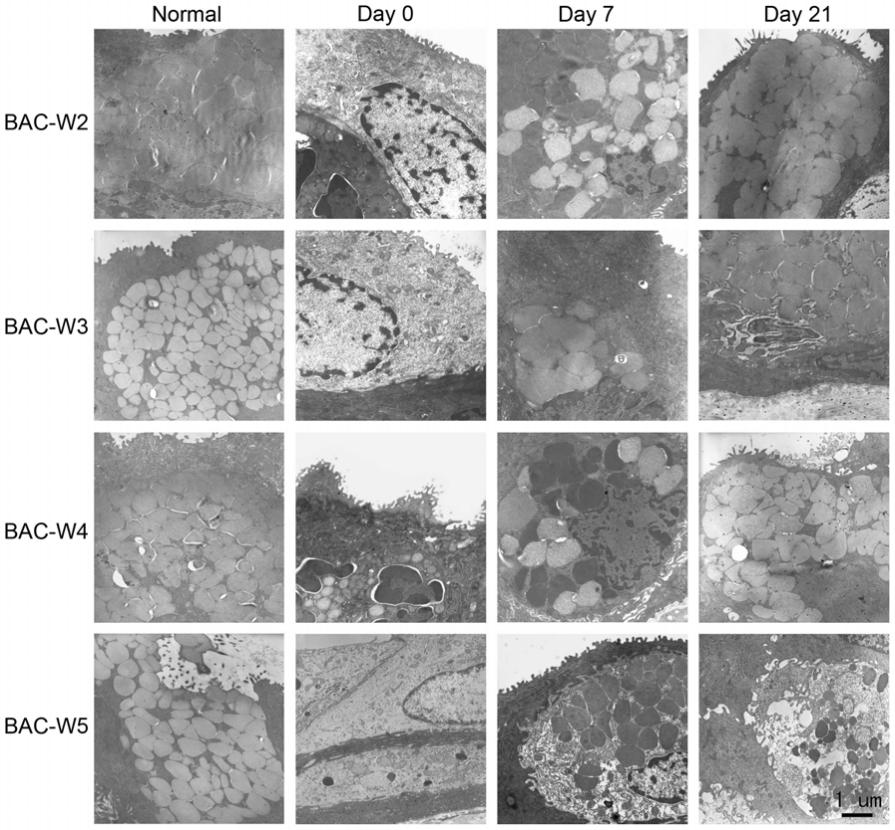
Representative images from transmission electron microscopy showing the conjunctival epithelial ultrastructure. Compared with normal eyes, fewer goblet cells and secretary granules were observed on day 0 in Groups BAC-W2 and BAC-W3, and on days 0, 7, and 21 in Group BAC-W4. This loss of goblet cells and secretary granules was particularly pronounced in Group BAC-W5 on all of the days that were studied. Chromatin condensation and peripheral migration could be observed on days 0, 7, and 21 in Group BAC-W5.

In Group BAC-W5, a specific number of secretory granules contained in the goblet cells could be observed on day 7, and the conjunctival epithelium appeared to have a more regular form that indicated that it was in an improved state. It is worth noting, however, that the damage that had been caused by regular applications of BAC remained severe in Group BAC-W5 on day 21; most of the goblet cells had no obvious secretory granules, and even dead goblet cells could be observed (Fig. 10).

## Discussion

Inflammation and increased tear osmolarity have been proposed as specific new additions to the definition of the dry eye disease that was reported by the Dry Eye WorkShop (DEWS) because the research regarding dry eye disease that has been conducted in recent years shows that the core pathogenesis mechanisms of dry eyes included heightened tear osmolarity and an unstable tear film [5]. According to this mechanism of pathogenesis, some symptoms of dry eye disease are due to increased tear osmolarity that arises from various causes and that can spur the ocular surface epithelial cells to produce and release inflammatory mediators into the tear film. This in turn leads to an inflammatory cascade that results in apoptosis of the ocular surface epithelial cells, and reductions in goblet cell density reducing [12], [24]. Another hypothesis regarding the pathogenesis of dry eye is one in which an unstable tear film that could be mainly caused by a mucin deficiency on the ocular surface is the initial problem, which implies that an increase tear osmolarity is not responsible for the onset of dry eye symptoms. Clinical examples of the latter include dry eye that results from the local administration of drugs that contain BAC [25], and these provide an important clinical basis of establishing this dry eye model. BAC is capable of destroying the tight junctions that are located in the superficial cells of the corneal epithelium [26]; this degradation of tight junctions then damages the normal functioning of the corneal epithelium [27]. Exposure to BAC can also lead directly to both apoptosis of the conjunctival epithelium [28], [29] and a decrease in the number of goblet cells [30]. Thus, as previous research has shown, the tear film becomes unstable and ceases to play its role in protecting the ocular surface [31]. In addition to having this effect on the cells themselves, BAC can stimulate the overexpression of inflammatory mediators in the epithelium, such as ICAM-1, or interleukins IL-6, IL-8, and IL-10 [32], [33], which may also cause apoptosis of both epithelial cells and goblet cells. The loss of these cells then leads to a reduction in mucin expression [34], [35]. These studies shows that in addition to the direct effect that BAC has on goblet cell density, inflammation underlies decreases in mucin expression. Reductions in the production of mucin speed up the rate at which the tear film breaks up, thereby aggravating the ocular surface damage and stimulating the inflammation cascade of the ocular surface epithelial cells. Ultimately, this results in a vicious cycle based on the core mechanisms of ocular surface protection that eventually leads to the occurrence of dry eye disease. A previous study demonstrated that the damage to the ocular surface that resulted from BAC administration was both dose- and time-dependent. The expression levels of apoptotic markers increased, and corneal and conjunctival cells were damaged immediately after in vitro exposure to a solution containing 0.1% BAC [35]. BAC has a high affinity for binding to ocular cells, and it can combine with the cellular plasma membranes quickly and persistently, which then results in a series of cellular alterations. At the same time, one drop of BAC can remain on the ocular surface for as long as 48 hours [36]. Furthermore, the topical application of a 0.1% solution of BAC did not cause severe damage to the ocular surface, such as ulcerations, large epithelial defects, or neovascularization. We had already constructed a rabbit dry eye model using a topical application of a 0.1% solution of BAC that was administered for 2 weeks [17]. In follow-up experiments that were based on this model, we found that the animal model that was established in this manner lacked stability, which was probably because the administration period was not long enough to cause permanent changes, so the eye that experienced the symptoms of dry eye was able to recover quickly. To obtain a stable animal model of dry eye that could be used for subsequent research, we designed the present experiment. We repeated the previous procedures that had been used to generate various models. After the cessation of BAC treatment, we observed both the self-recovery of the ocular conditions of the experimental animals and some related indices. Our results showed that the values of the indices that we measured in each experimental group on day 0 after we stopped administering the medication were consistent with the characteristics of dry eye. This finding also agreed with the results of our previous study [17] and those of studies that were conducted by other researchers [10], [15]. However, although some indicators of dry eye persisted as the time over which we continued to observe the model lengthened, some indices of the dry eye model that we had established gradually recovered.

In the present study, the result of the Schirmer's test showed that in Group BAC-W5, the basic signs of reductions in tear secretion could remain for as long as 2 weeks after treatment with BAC was stopped; in other groups, these indicators generally returned to normal in the third week following the cessation of treatment. This finding suggests that the early reduction in basic tear secretion is probably due to direct damage of the accessory lacrimal glands and the conjunctiva.

The results of corneal fluorescein staining showed that changes in fluorescein staining, which reflects defects among the corneal epithelial cells, could be sustained for 2 weeks after the cessation of treatment in all of the rabbits, and that fluorescein staining returned to normal levels on day 21. Combining this result with the results of scanning and transmission electron microscopy, we found that the BAC-induced damage to the corneal and conjunctival ultrastructure could last for at least 21 days in Group BAC-W5. This result revealed that although there were few obvious defects in the corneal epithelial cells in Group BAC-W5 animals on day 21, damage to the corneal and conjunctival ultrastructure may be secondary to inflammation that was stimulated by BAC. This damage included facets such as the shortening and lodging of microvilli on the corneal surface and the reduction in micropores due to changes in cell metabolism.

MUC5AC is the most essential subtype of the family of ocular surface mucins [37], and it is mainly secreted by the goblet cells. In this study, the trends in the changes in MUC5AC immunofluorescence in each group were consistent with the results of PAS staining. MUC5AC production seemed to be recovering within two weeks of the cessation of BAC treatments, but the expression levels were still significantly reduced compared with normal levels. This represents a recovery of the goblet cells due to the removal of the direct effect of BAC on the ocular surface. However, the numbers of goblet cells and the amounts of MUC5AC in Group BAC-W5 specimens remained at significantly lower levels that were as low on day 21 as they were on day 0. This behavior resembled that which had been observed using transmission electron microscopy, which showed that on day 21, the reduction in the number of secretory granules in conjunctival goblet cells in Group BAC-W5 was still significant compared with the number of secretory granules in normal tissue. This could be because the topical application of 0.1% BAC for 5 weeks resulted in a stable inflammatory immune environment, in which the epithelium was stimulated to express inflammatory mediators that then caused goblet cell apoptosis and an inability to return to normal levels of secretory granule production [34], [35], [38], despite the lack of a direct effect of BAC on the goblet cells. The continued insufficiency of MUC5AC production then resulted in an unstable tear film [39], which in turn caused serial ocular damage and an inflammatory environment. Thus, the eye entered a vicious cycle that ultimately led to the development of dry eye. Therefore, we think that the animal model in which dry eye is induced by 5 weeks of topical administration of 0.1% BAC is a stable model of mucin deficiency that is associated with dry eye.

In this paper, we demonstrated that topical administration of BAC for a period of 2–4 weeks could successfully produce an animal model of dry eye that was both satisfactory and applicable. This model could be used to study both the prevention and the etiopathogenisis of dry eye, or it could be expanded by means of some appropriate interventional measures in the modeling process. A stable animal model of dry eye can be induced via a 5-week course of topical administration of BAC. A water-deficient state that results from this model could last 2 weeks, and it could be used for research regarding the water-deficient subtype of dry eye. It is worth noting that including a control group that is based on the model and for which the observation time is longer than the current temporal window would make our results more credible. Both the number of goblet cells and the secretory volume of MUC5AC remained at low levels for 3 weeks after dry eye was induced in the model animals; the diminished secretion state in the goblet cells did not display any recovery after BAC was no longer administered. All of the above phenomena demonstrate that the topical administration of a 0.1% solution of BAC for 5 weeks established a stable model of dry eye that included an extended period over which the secretion of mucin proteins was diminished relative to baseline levels. Due to the stability of this model and the low levels of mucin secretion that it produces, this rabbit model might be particularly appropriate for research regarding the etiology and pathogenesis of dry eye and for research regarding the therapeutic effects of medication and other treatments for dry eye that is accompanied by mucin deficiency or epithelial damage.
