# Correction: Research on the Stability of a Rabbit Dry Eye Model Induced by Topical Application of the Preservative Benzalkonium Chloride

**DOI:** 10.1371/annotation/f2df1a21-1df3-4c2d-a99a-b054dfbc6443

**Published:** 2012-07-10

**Authors:** Chaoyang Li, Yiyue Song, Shaohong Luan, Pengxia Wan, Naiyang Li, Jing Tang, Yu Han, Cuiju Xiong, Zhichong Wang

There was an error in the funding statement. The correct funding statement is: "This research was supported by grant No. 30973246 (W.Z.C.) from the National Natural Science Foundation of China (http://www.nsfc.gov.cn); [^] grant No. 2009A030200004 (W.Z.C.) from the Science and Technology Planning Project of Guangdong Province, China (http://www.gdstc.gov.cn); [^] grant No. B2011106 (W.P.X.) from the Medical Science Foundation of Guangdong Province (http://www.gdwst.gov.cn); [^] grant No. S2011040004327 (W.P.X.) from the Doctor Initiating Project of the National Science Foundation of Guangdong Province; and the Fundamental Research Funds of the State Key Laboratory of Ophthalmology. The funders had no role in the study design, data collection and analysis, decision to publish, or preparation of this manuscript." 

